# Chinese social media reaction to the MERS-CoV and avian influenza A(H7N9) outbreaks

**DOI:** 10.1186/2049-9957-2-31

**Published:** 2013-12-20

**Authors:** Isaac Chun-Hai Fung, King-Wa Fu, Yuchen Ying, Braydon Schaible, Yi Hao, Chung-Hong Chan, Zion Tsz-Ho Tse

**Affiliations:** 1 Department of Epidemiology, Jiann-Ping Hsu College of Public Health, Georgia Southern University, Statesboro, Georgia, USA; 2 Journalism and Media Studies Center, The University of Hong Kong, Hong Kong Special Administrative Region, China; 3 Department of Computer Science, The University of Georgia, Athens, Georgia, USA; 4 Department of Biostatistics, Jiann-Ping Hsu College of Public Health, Georgia Southern University, Statesboro, Georgia, USA; 5 College of Engineering, The University of Georgia, Athens, Georgia, USA

## Abstract

**Background:**

As internet and social media use have skyrocketed, epidemiologists have begun to use online data such as Google query data and Twitter trends to track the activity levels of influenza and other infectious diseases. In China, Weibo is an extremely popular microblogging site that is equivalent to Twitter. Capitalizing on the wealth of public opinion data contained in posts on Weibo, this study used Weibo as a measure of the Chinese people’s reactions to two different outbreaks: the 2012 Middle East Respiratory Syndrome Coronavirus (MERS-CoV) outbreak, and the 2013 outbreak of human infection of avian influenza A(H7N9) in China.

**Methods:**

Keyword searches were performed in Weibo data collected by The University of Hong Kong’s Weiboscope project. Baseline values were determined for each keyword and reaction values per million posts in the days after outbreak information was released to the public.

**Results:**

The results show that the Chinese people reacted significantly to both outbreaks online, where their social media reaction was two orders of magnitude stronger to the H7N9 influenza outbreak that happened in China than the MERS-CoV outbreak that was far away from China.

**Conclusions:**

These results demonstrate that social media could be a useful measure of public awareness and reaction to disease outbreak information released by health authorities.

## Multilingual abstract

Please see Additional file 1 for translations of the abstract into the six official working languages of the United Nations.

## Background

Digital epidemiology is a quickly growing field that uses digital (e.g. Internet) information to study the distribution of diseases and other health conditions over time and in different geographical areas [1,2]. Various online data have been harnessed for public health surveillance purposes [3]. For example, search engine query data from Google have been used to estimate weekly influenza activity in a number of countries (Google Flu Trends) [4] and Google query data in French were correlated with French surveillance data for influenza, acute diarrhea and chickenpox [5]. Search engine query data from other search engines, namely Yahoo and Baidu, also correlated well with influenza surveillance data in the US and China, respectively [6,7]. Online news data from HealthMap [8] were used to track the 2010 Haitian cholera outbreak, along with social media data (Twitter) [9].

Social media data could be harnessed to analyze the public's concern about an infectious disease outbreak. Scientists studied Twitter data to monitor influenza activity [10], public concern about H1N1 influenza [11,12], and sentiments about H1N1 influenza vaccination [13]. Algorithms were developed to distinguish tweets that mentioned someone’s experiences with influenza from those that expressed worries about it [14]. The 2013 H7N9 influenza outbreak in China also drew the attention of epidemiologists toward the potential ability to monitor disease outbreaks using digital data [15].

*Weibo*, translated “microblog”, is the Chinese social media equivalent to Twitter. Like Twitter, Weibo allows users to post and share messages carrying at most 140 Chinese characters. Users may optionally attach links, images, or videos to their messages. Weibo also allows users to “follow” others’ Weibo accounts (“friends”) or to repost (or “retweet”, in Twitter parlance) another user's posts to one's own readership (“followers”). Despite the government’s control on the Internet content [16], Weibo still enables Chinese people to publish messages about public incidents or disseminate information during natural disasters [17]. It was described by Western media as a new “free speech platform” [18]. One major Weibo service provider in China, Sina Weibo, claimed to have over 500 million registered users at the end of 2012 [19].

Our study is the first to use Chinese social media (Weibo) data to study the Chinese online community’s reaction to the release of official outbreak data from health authorities, namely the outbreaks of MERS-CoV in 2012 [20] and of human infections of avian influenza A(H7N9) in 2013 [21,22]. Our hypothesis was that China's online community would have a stronger reaction to an outbreak in China than one outside China. Our analysis allows health authorities and the media to better understand the online dynamics of health communications in outbreak scenarios.

## Methods

### Data acquisition and sampling

Weibo data were collected by The University of Hong Kong’s Weiboscope project. The project’s primary aim is to develop a data collection and visualization system for better understanding of Weibo in China. Details of the methodology have been reported elsewhere [16]. In summary, the project generated a list of about 350,000 indexed microbloggers by searching the Sina Weibo user database systematically using the Application Programming Interface (API) functions provided by Sina Weibo. The inclusion criterion was those users who have at least 1,000 followers. We used high-follower-count samples for two reasons: first, in social media, high-follower-count users are relatively more influential and can often draw disproportionally larger public attention [23]. Second, this sampling strategy can minimize the influence of spam accounts, which were found widespread in China’s social media [24]. Because of the heightened restriction on Sina Weibo API access, the microbloggers included in the data acquisition since January 2013 were restricted to a selective group of around 50,000 “opinion leaders” with at least 10,000 followers. This group of microbloggers was selected for analysis in the current study in order to have fair comparison between the keyword frequencies in 2012 and 2013.

For each indexed microblogger on the list, all new Weibo messages posted were fetched periodically by using Sina Weibo’s user timeline API function. Newly collected messages were cached in the database for future data analysis. The frequency of revisiting the user timeline of the indexed microbloggers varied from every three minutes to once monthly, which depended on multiple factors that were chosen to maximize detection of each user’s posts [16] while making efficient use of the per-hour API rate limit imposed by Sina Weibo as well as our limited computing resources (See Additional file 2 – Appendix for more details).

### Keyword detection and data analysis

The Weibo raw data was acquired over the period of January 1, 2012 to June 30, 2013 in Comma-Separated Values (CSV) format and sorted by week [16]. The CSV files contain useful metadata available for analysis, including the Weibo posts, the created date and user ID data. The user IDs were “hashed” before storing them, meaning they were converted into a different string of characters so that the user ID is not directly displayed in the database. The first line of each file describes the properties of the file, followed by the Weibo post record.

**Figure 1:**
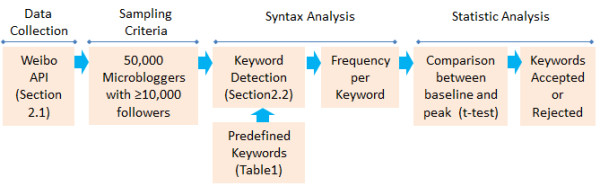
Workflow for keyword selection and analysis.

Keyword detection started with a simple string-searching algorithm; given a keyword of a particular disease, for example, H7N9, the algorithm searched every Weibo post and recorded if and how many times the particular keyword appeared in the data file. Table 1 shows the list of keywords that were used in the searching process and were included in the final analysis. Figure 1 shows the workflow for keyword selection and analysis. Figure S1 in Additional file 2 – Appendix shows the flowchart of the Keyword Detection Scheme. Please refer to Additional file 2 – Appendix for more details.

**Table 1:** Keywords used in Weibo post search of which the results were kept in the final analysis of this study

**Keywords**	**Transliteration in English under the Hanyu Pinyin system**	**Description**
禽流感*	qín liúgǎn	Avian Influenza (Avian Flu)
H7N9	N/A	H7N9 refers to the type of hemagglutinin and neuraminidase of the influenza strain. This is used as part of the standard nomenclature of influenza strain
SARS	N/A	The acronym for Severe Acute Respiratory Syndrome
沙士	sà sī	The phonetic transliteration of SARS
冠状病毒	guàn zhuàng bìng dú	Coronavirus

*流感 (liúgǎn; flu) is the short form of 流行性感冒 (liú xíng xìng gǎn mào; influenza), just like flu is the short form for influenza in English.

We used official press releases of outbreak data by WHO and the Chinese government as "signals" (or the assumed sources of outbreak news) to which the Chinese online community reacted. The Global Alert and Response press release by WHO on September 23, 2012 was used as a "signal" for news on MERS-CoV (then known as "a novel coronavirus") [20], and the March 31, 2013 press release by the Chinese National Health and Family Planning Commission was used as a "signal" for news on human infections of avian influenza A(H7N9) [22].

Statistical analysis was performed using Microsoft Excel, SAS 9.3 Base and R 2.15.3. We first established the baseline for each keyword and then measured the online response (both magnitude and time to peak) compared to the baseline. We normalized the number of posts with a particular keyword on a given day by dividing it by the total number of posts in our sample for that day, and then multiplying it by 1,000,000 to obtain the number of tweets with a particular keyword per 1 million tweets. The 2012 data (January 3 - December 30) was used to establish the baseline data for Weibo posts with keywords "avian flu" and "H7N9". Likewise, part of the 2012 data, prior to September 23, 2012, was used to establish the baseline for the keywords that were related to MERS-CoV. We chose 2012 as the baseline year, assuming that the underlying Weibo conversations about health-related information were not significantly different between 2012 and 2013. One-sample t-test (two-sided) was used to measure the statistical significance of the difference between the peaks and their corresponding baseline values.

A new website dedicated to this project, named WeiboHealth [25], was created to share our updated results with public health researchers and practitioners.

## Results

### Human infections of avian influenza A(H7N9), March - April 2013

**Figure 2:**
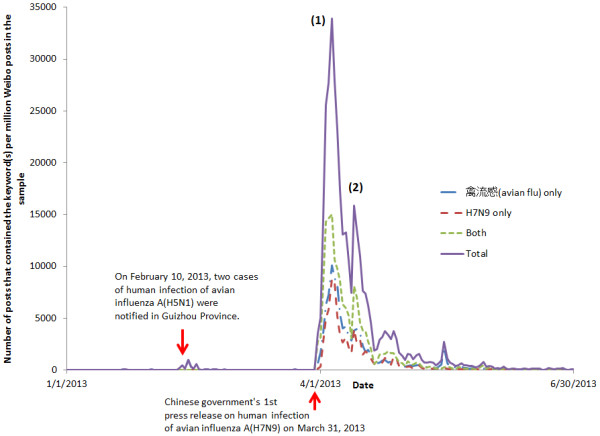
**Chinese online community's reaction to Chinese government's press releases on avian influenza A(H7N9) in 2013.** The daily numbers of Weibo posts that contain “H7N9”, “avian flu”, or both per million posts in the sample of about 50,000 users that have ≥10,000 followers, from January 1 to June 30, 2013, are shown here. Notes: 1) The volume of H7N9-related Weibo posts reached its first peak on April 5, 2013, five days after the first press release of the Chinese government on human infection of avian influenza A (H7N9); 2) a second peak was observed on April 13, 2013, the day when the Beijing municipal authorities announced that one case was diagnosed as H7N9-positive in Beijing.

The reaction to the news of human infection of avian influenza A(H7N9) was very profound in the Chinese online community. Among the users with ≥10,000 followers, a peak of 33,904 per million Weibo posts (t = −20,836; p < 0.001) that contain the keywords "禽流感" (Qinliugan in pinyin, a Mandarin Chinese phonetic script, avian flu) or "H7N9" or both was observed on April 5, 2013, five days after the Chinese government press release on March 31, 2013. This was 1093.6 times the standard deviation (s.d.) away from the mean of the baseline value in 2012 (mean, 24.19; s.d., 30.98) (Table 2). After the peak, there was a quick decline in Weibo discussion on this topic. The number of Weibo posts that contain "H7N9" and/or "禽流感" (avian flu) declined to 7,469 per million on April 12 (a decline of 3,638.7 posts per day from April 5 to 12, assuming a linear trend, R^2^ = 0.9433). On April 13, the Chinese National Health and Family Planning Commission announced that there was a H7N9-positive case in Beijing. The H7N9 avian flu-related posts doubled (15,864 per million, t = −9,741; p < 0.001). After this second peak, the attention waned and the number of posts on H7N9 avian flu declined at a rate of 1,873.6 per million per day to 1,883 per million on April 20, 2013 (Figure 2). If only the keyword "H7N9" was used, the signal was even more sensitive. Given its very low baseline in 2012 (mean, 0.027 per million posts, s.d. 0.265), its peak of 8,803 per million posts (t = −632,933; p < 0.001) was 33,220 s.d. away from the baseline mean.

**Table 2:** Chinese social media’s reaction to the early reports of the influenza A(H7N9) outbreak

	2012 Baseline* (mean and s.d.)	**Peak value on April 5, 2013**	**Number of s.d. the peak is away from the baseline mean**
"禽流感" (Qinliugan, avian flu) only	24.19 (s.d. 30.98)	10,113	326†
"H7N9" only	0.027 (s.d. 0.265)	8,803	33220†
"禽流感" AND "H7N9"	0	14,988	n/a
Total	24.22 (s.d. 30.98)	33,904	1094†

*Baseline data were based on data from January 3 to December 30, 2012. We did not use the data for Jan 1–2, 2012 because of a peak for "禽流感" (Qinliugan, avian flu) on Jan 2, 2012 that was considered as an outlier. That peak was a result of the news released on that day about a patient who died of highly pathogenic avian influenza in Shenzhen, Guangdong Province, China, on December 31, 2011 [26]. Unit: per million posts. †p < 0.001. s.d., standard deviation.

Baseline and peak values are presented as number per million Weibo posts that contain keywords for avian flu and H7N9 in our samples of about 50,000 users with ≥10,000 followers, in 2012 and 2013.

In our pilot studies, we had also tried the keywords “流行性感冒” (liúxíngxìng gǎnmào; influenza) and “流感” (liúgǎn; short form for liúxíngxìng gǎnmào flu; English equivalent: flu). For the former, few posts (per day) contained this formal technical term, and so we decided to drop it in further analysis (data not shown). For the latter, since the keyword “禽流感” (avian flu) is more specific and it actually contained the term “流感” (flu), we decided to use “禽流感” (avian flu) in our analysis instead of “流感” (flu) (data not shown).

### MERS-CoV, September 2012

**Figure 3:**
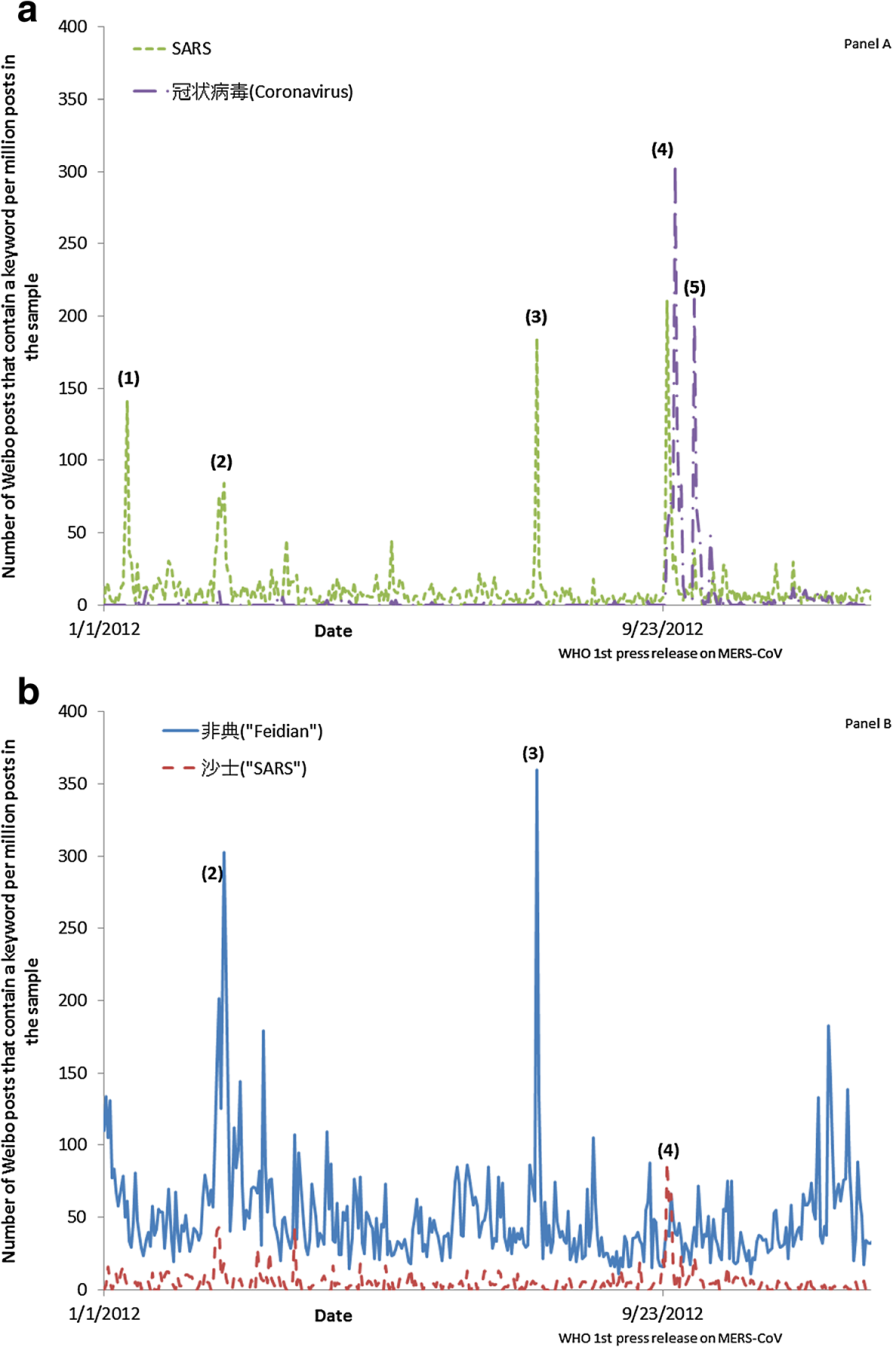
**Chinese online community's discussion related to SARS in 2012 and its reaction to WHO's 1st press release on MERS-CoV on September 23, 2012.** Panel **(a)** Keywords: “SARS”; “冠状病毒” (coronavirus). Panel **(b)** Keywords: “非典” (Feidian); “沙士” (SARS). The daily numbers of posts that contain a keyword per million posts in the sample of about 50,000 users that have ≥10,000 followers, from January 1 to December 31, 2012, are shown here. For Weibo posts that have more than one keyword, they were grouped under the first keyword in the post. This figure shows that while the keywords “SARS”, “冠状病毒” (coronavirus) or “沙士” (SARS), were sensitive to the news of MERS-CoV (peak 3), “非典” (Feidian, short for fei-dianxing-feiyan, translated, “atypical pneumonia,” is the layman’s term for SARS in China) is not.^a^

The Chinese online community also reacted to the news of a novel coronavirus, now known as MERS-CoV, identified in a patient in the UK, but in a less pronounced way (Figure 3; Table 3).

**Table 3:** Chinese social media’s reaction to the first WHO report of MERS-CoV outbreak

	Baseline mean (s.d.) (time period*)	**Number per million Weibo posts that contain a given keyword on a given day (number of s.d. away from the mean)**								
		**Sep 22**	**Sep 23**	**Sep 24**	**Sep 25**	**Sep 26**	**Sep 27**	**Sep 28**	**Sep 29**	**Sep 30**
非典 (Feidian, shortened for atypical pneumonia)	46.95 (s.d., 20.62) (Apr 1 - Jul 1)	16.3 (−1.44)	15.6 (−1.47)	32.7 (−0.64)	40.3 (−0.27)	36.5 (−0.46)	66.2 (0.98)	49.4 (0.17)	39.0 (−0.34)	37.2 (−0.42)
沙士 (phonetic translation of SARS)	3.97(s.d., 3.84) (May 1 - Sep 1)	2.7 (−0.33)	20.8 (4.39)	14.0 (2.62)	87.4 (21.75)	58.3 (14.17)	66.2 (16.22)	15.7 (3.06)	0 (−1.04)	8.0 (1.04)
SARS	7.81 (s.d., 6.59) (Apr 1 - Jul 1)	2.7 (−0.77)	5.2 (−0.40)	32.7 (3.78)	210.7 (30.80)	119.1 (16.89)	75.3 (10.25)	24.7 (2.56)	33.4 (3.88)	13.3 (0.83)
冠状病毒 (coronavirus)	0.33 (s.d., 22.74) (Jan 1 - Sep 1)	0 (−0.01)	0 (−0.01)	0 (−0.01)	51.6 (2.25)	63.2 (2.76)	70.8 (3.10)	65.1 (2.85)	306.3 (13.45)	125.0 (5.48)

Note: The term "Middle East Respiratory Syndrome" had not been invented in 2012. Most users of Weibo would use terminology related to SARS to refer to the novel coronavirus that was renamed Middle East Respiratory Syndrome-Coronavirus (MERS-CoV) in 2013. *We chose a different time period as the baseline to avoid outliers related to other events in the news that would bias the data. SARS, Severe Acute Respiratory Syndrome; s.d., standard deviation.

Baseline and peak values are presented as number per million Weibo posts that contain keywords related to MERS-CoV, namely keywords of SARS and coronavirus in our sample of about 50,000 users with ≥10,000 followers, in 2012. This table shows that three of the four keywords were sensitive to the news of the novel coronavirus causing a disease similar to SARS.

Nine different keywords that were related to SARS were tested, and three of them were found both sensitive and specific enough to reflect the Chinese online community's reaction to this novel coronavirus (Table 1). On September 23, 2012 when WHO released its press release on the novel coronavirus, the number of Weibo posts about “沙士” (SARS), posted by the ~50,000 users with ≥10,000 followers, increased to 20.8 per million (4.4 s.d. away from the baseline mean; t = −49, p < 0.001) and two days later, it rose to 87.4 per million (21.8 s.d. away; t = −242, p < 0.001) (Figure 3b) For Weibo posts mentioning the English acronym SARS, they reached a peak of 210.7 per million (30.8 s.d. away; t = −295, p < 0.001) on September 25, 2012 (Figure 3a). For Weibo posts carrying the virological term "冠状病毒" (guàn zhuàng bìng dú, Coronavirus), it rose from 0 to 51.6 per million posts (2.25 s.d. away; t = −35, p < 0.001) on September 25, 2012, and continued to rise to a peak of 306.3 per million posts (13.5 s.d. away; t = −21, p < 0.001) on September 29, 2012 (Figure 3a). The official translation of Severe Acute Respiratory Syndrome was never found in our sample in 2012. Three other phonetic translations of SARS as well as two renditions of atypical pneumonia were either not sensitive or non-specific to the WHO press release on MERS-CoV on September 23, 2012 (Table 4).

**Table 4:** Keywords about SARS that were either insensitive or non-specific to the news of MERS-CoV on September 23, 2012

**Keywords in simplified Chinese**	**Transliteration in English under the Hanyu Pinyin system**	**Description**	**Reason for exclusion**	**Explanation**
严重急性呼吸综合症	yán zhòng jí xìng hū xī zōng hé zhēng	The official/technical translation of the medical term "Severe Acute Respiratory Syndrome"	Not sensitive	In our sample in 2012, not a single post mentioned this technical term.
萨斯	sà sī	One of the four phonetic transliterations of SARS	Not specific	The result is very noisy and therefore this term is not specific to the signal.
沙斯	sà sī	One of the four phonetic transliterations of SARS	Too few posts and not sensitive	No reaction to the WHO press release on MERS on September 23, 2012 was detected.
煞斯	sà sī	One of the four phonetic transliterations of SARS	Too few posts and not sensitive	No reaction to the WHO press release on MERS on September 23, 2012 was detected.
非典型肺炎	fēi diǎn xíng fèi yán	Atypical pneumonia	Too few posts and not sensitive	No reaction to the WHO press release on MERS on September 23, 2012 was detected. The absolute number of Weibo posts that mentioned this keyword was so small that it was rendered subject to the influence of noise.
非典	fēi diǎn	Short form for atypical pneumonia	Not sensitive	No reaction to the WHO press release on MERS on September 23, 2012 was detected.

### SARS-related posts during the H7N9 outbreak, 2013

**Figure 4:**
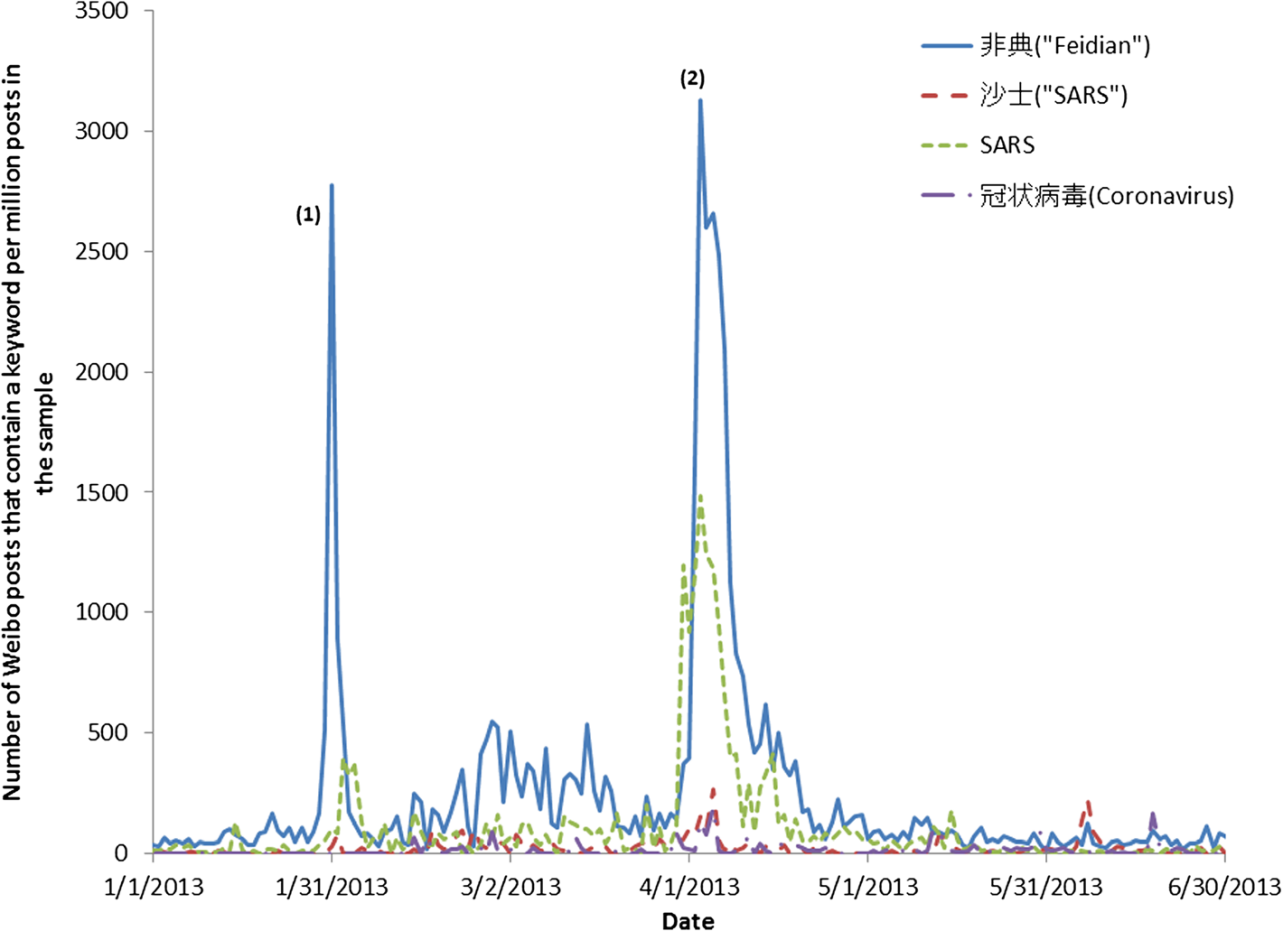
**Chinese online community's discussion related to SARS, January - June 2013.** The daily numbers of posts that contain a keyword per million posts in the sample of about 50,000 users that have ≥10,000 followers, January 1 to June 30, 2013 are shown here. 非典, (Feidian, short for fei-dianxing-feiyan, translated, “atypical pneumonia”) is the layman’s term for SARS in China. Notes: (1) On January 31, 2013, in an interview with the media, Prof. ZHONG Nan-Shan, a famous Chinese medical expert with a high reputation from his experience fighting against SARS in 2003, mentioned that air pollution in China is more dreadful than “Feidian” because no one can escape from it. His quote from the interview was re-posted widely by Weibo users on that day. (2) Beginning on March 31, 2013, Weibo posts with keywords “Feidian” or SARS skyrocketed, and reached a peak on April 3, 2013. Likewise, Weibo posts with keywords “沙士” (SARS) or “冠状病毒” increased, and reached a peak on April 5, 2013.

We also studied how the traffic of Weibo posts carrying SARS-related keywords reacted to the H7N9 outbreak. Beginning on March 31, 2013, Weibo posts with keywords “非典” (Feidian, shortened for atypical pneumonia) or the English acronym SARS rocketed, and reached a peak on April 3, 2013. Likewise, Weibo posts with keywords “沙士” (SARS) or “冠状病毒” (Coronavirus) increased, and reached a peak on April 5, 2013 (Figure 4).

### Comparison

We observed that the strength of the reaction to the H7N9 outbreak (peak: 33,904 posts per million posts on April 5, 2013; keywords “禽流感” (avian flu) and “H7N9”) was two orders of magnitude stronger than the reaction to the MERS-CoV outbreak (peak: 349 posts per million posts on September 25, 2013; keywords: “沙士” (SARS), SARS, and “冠状病毒” (Coronavirus)) (Figures 2 and 3).

## Discussion

The Chinese online community reacted rapidly to news about infectious disease outbreaks both within and beyond China, as shown in our study. This paper is the first to document this online response using Weibo and to compare the reaction to the MERS-CoV outbreak in 2012 with the reaction to the human infections of avian influenza A(H7N9) in 2013. We found that the reaction to the H7N9 outbreak in 2013 was about two orders of magnitude stronger than the one to the MERS-CoV outbreak in 2012. The results confirmed our hypothesis that the Chinese online community reacted more strongly to an outbreak that was in China than one outside China.

The reaction in the Chinese online community exploded within the first five days of the first case report of three human cases (two in Shanghai and one in Anhui) of avian influenza A(H7N9) [22]. Within these five days, more cases were identified in Shanghai and in two neighboring provinces of Jiangsu and Zhejiang. However, attention soon declined rapidly. It declined until April 13, 2013, when the Chinese government announced that a child was found H7N9-positive in Beijing, the capital of China. This piece of news triggered a second explosion of online discussion via Weibo on that day. Attention then declined rapidly again (Figure 2).

Keywords that were sensitive and specific to the signals were identified. Keywords like "H7N9" and "冠状病毒" (Coronavirus) were highly sensitive and specific. Keywords like "禽流感" (avian flu) and SARS, while less specific, remained sensitive enough to detect the signals.

While the keyword "非典" (Feidian, shortened for atypical pneumonia) was not sensitive to the news of MERS-CoV on September 23, 2012 (Figure 3b), we would like to highlight its significance in the lexicon of the current Chinese online community as one of its most frequently used term for SARS in online discussion. As a keyword, "非典" (Feidian) was sensitive to rumors of SARS in the city of Baoding, China, on February 19, 2012. The rumors were later rejected by the Chinese authorities on February 26, 2012 when the possibility of SARS infection among feverish hospitalized patients in a hospital in Baoding was excluded (Figure 3b) [27]. This keyword, however, also led to a "false positive". On July 21, 2012, there was a severe flood in Beijing, resulting in dozens of deaths. The Chinese online community complained about the Beijing municipal government's disaster management. The government reacted by holding a press conference on July 24, saying that they had learned the lessons of SARS in 2003 and did not conceal the true death toll [28]. This incident also led to a peak in posts with the keyword "非典" (Feidian) (Figure 3b). On January 30, 2013, in a telephone interview with the China Central Television, Prof. ZHONG Nan-Shan, a well-respected medical researcher with a reputation as a leader in fighting against SARS in 2003 in China, mentioned that air pollution in China was more dreadful than "非典" (Feidian) because no one could escape from it [29]. His quote from the interview also led to a peak of Weibo posts with the keyword "非典" (Feidian) (Figure 4).

The observation that Weibo posts with the keywords "非典" (Feidian) and SARS rose to 3131.9 and 1485.4 per million on April 3, 2013 (Figure 4) was consistent with a similar observation in web search query data from Google Trends ([30]; search terms: SARS; "非典"; time range: 2013; Location: China; accessed on October 5, 2013), in which a peak was observed during the week of March 31, 2013. Given China’s SARS experience in 2003, the Chinese online community’s reaction is not surprising. Our observations show that the Chinese online community discussed SARS in the first week after the first report of the H7N9 outbreak with an order of magnitude higher frequency than that in the first week after the first report of the MERS-CoV outbreak. These results again confirmed our hypothesis that the Chinese online community reacted more strongly to an outbreak that happened in China than one outside China.

Drawing on the social amplification of risk model [31], public risk perception is shaped by a process of interplays between psychological, cultural, social, and institutional factors that may result in amplifying or attenuating the public attention to risk. Mass communication is among the list of factors. Public health officials have long recognised the role of the mass media in disseminating risk and emergency information before, during, and after a catastrophe [32]. The World Health Organization establishes guidelines for “effective media communication”, through which the authorities are able to disseminate information to the public [33]. Communication during crisis was traditionally understood to be a one-way and top-down process, in which the public are assumed to be “deficient” in knowledge, while the scientists, public health experts, and emergency managers, are “sufficient” [34]. But this presumption was profoundly challenged by the emergence of social media. For instance, Leung and Nicoll argued that the 2009 H1N1 pandemic was the first pandemic in which social media “challenged conventional public health communication” [35]. In China, online messages were published ahead of the official statement in the 2008 Sichuan Earthquake [36]. Social media enabled people under crisis to share information and experience and to seek message credibility and confirmation via multiple media platforms and social networks [34]. Our study demonstrated that official data released by health authorities, whether in Beijing or Geneva, received strong reactions in the Chinese online community. With such knowledge, social media should be incorporated in the best practices for risk and crisis communication [37]. Social media data can also provide health authorities, researchers and the media a quantifiable measure of public attention towards a particular disease outbreak [11].

Social media, in addition to being a tool to release and track official outbreak information [38], offers a new opportunity for public health practitioners to understand social and behavioral barriers to infection control, to identify misinformation and emerging rumors [39], and to better understand the sentiments and risk perception associated with outbreaks and preventive and control measures [13]. In turn, these will help facilitate better health communication between public health agencies and the society at large, as well as among citizens themselves.

With our Weibo data, there are at least two potential directions for future research. First, we can study how information about a given disease spread across the social network as represented by Weibo. Kwak et al. [40] identified a non-power-law follower distribution, a short effective diameter and low reciprocity in Twitter follower-following topology, which was different from most human social networks. Over 85% of the top trending topics on Twitter are headline news or persistent news. Once retweeted, a tweet would reach an average of 1,000 users regardless of original tweet’s number of followers [40]. However, a previous study has found that Chinese Weibo exhibits a distinct pattern of information dissemination [41]. For example, the network connections between Chinese microbloggers are markedly hierarchical than those between Twitter users, i.e. Chinese users tend to follow those at a higher or similar social level [42]; majority of Weibo posts are indeed re-posts that are originated from a small percentage of original messages [24]. It will be very interesting if further research can shed light upon how information sharing over Weibo can affect human response to the diseases off-line.

Second, content analysis of Weibo posts will enable us to analyze human attitudes or reactions toward health hazard [43]. The research can be extended to investigate anxiety or fear towards the infectious diseases themselves and towards the outbreak information transmitted via the Weibo social network. Similar research on influenza has been conducted using Twitter data [12,14]. Data mining methods, like topic models [44], may be attempted.

There are a few limitations to our study. The sampled microbloggers in our study were limited to those who have more than 10,000 followers. Despite the fact that these microbloggers are more likely to be authentic users rather than spam accounts, the samples constitute less than 0.1% of the overall microblogger population [23]. However, a random sampling study finds that Weibo content contribution is unevenly distributed among users [23]. Over half of Sina Weibo subscribers have never posted, whereas about 5% of Weibo users contributed more than 80% of the original posts [23]. Hence, the sampled microbloggers in our study were the most influential microbloggers who contributed a majority of Weibo posts and drew the most attention in terms of the number of reposts and comments [23]. Therefore, for the purpose of this study, this group of high-follower-count microbloggers should be deemed fairly representative of the public attention towards the MERS-CoV and H7N9 outbreaks. But the reader should note that the findings of our study might not be generalizable to the samples collected by other sampling strategies. The operational parameters of sampling were not determined to optimize collection of data specific to a given disease. Future research is warranted to reconfirm the research findings by using a research design that is customized for specific epidemiologic research purposes.

## Conclusion

This is the first paper that documents the online Chinese community's reaction to the MERS-CoV outbreak in the Middle East and Europe in 2012, as well as the reaction to the H7N9 outbreak in China in 2013. The reaction to H7N9 was two orders of magnitude stronger than the reaction to MERS-CoV. Similar to the public reaction on the street, the online community's reaction is stronger when the disease outbreak happens nearby. Our study demonstrates the usefulness of using social media to measure the public reaction to disease outbreak information released by health authorities.

### Endnote

^a^Notes on peaks in Figure 3: 1) The peak on January 12, 2012 was a false positive. None of the posts were genuinely related to “SARS”. 2) On February 19, 2012, rumors began to circulate that hospitalized patients in a hospital in the city of Baoding, China, were diagnosed with SARS. A week later (February 26), the Chinese authorities excluded the possibility of SARS among feverish hospitalized patients in that hospital. The volume of Weibo posts peaked on February 27. 3) On July 21, 2012, extremely heavy rain led to flooding in Beijing, resulting in many deaths and injuries. Responding to allegations that the government concealed the true death toll, the Beijing municipal government replied on July 24 that they had learned their lesson from the 2003 SARS outbreak and they would not conceal the truth. The volume of Weibo posts peaked on July 25. 4) After WHO 1st press release on MERS-CoV on September 23, 2013, Weibo posts with the keyword “SARS” reached its peak on September 25, 2013 while Weibo posts with the keyword “冠状病毒” (coronavirus) reached its peak on September 29, 2013. 5) On October 8, 2013, there was news about a probable case of MERS-CoV infection in Hong Kong. The probable case patient was a child from Saudi Arabia. The child was later confirmed of having influenza infection, instead of MERS-CoV. A peak of Weibo posts with the keyword “冠状病毒” (coronavirus) was found on that day, as the Chinese newscast of that day used the term “新型冠状病毒” (novel coronavirus) [45].

## Abbreviations

API: Application programming interface; CSV: Comma-separated values; MERS-CoV: Middle East respiratory syndrome-coronavirus; SARS: Severe acute respiratory syndrome.

## Competing interests

The authors declare that they have no competing interests.

## Authors’ contributions

ICHF, ZTHT and KWF conceived of the study, participated in its design and coordination and drafted the manuscript. CHC performed the data acquisition and sampling. YY performed the keyword detection and created the WeiboHealth website. Both CHC and YY helped draft parts of the manuscript. ICHF, BS and YH performed the data analysis. All authors read and approved the final manuscript.

## Authors’ information

ICHF is an assistant professor in the Department of Epidemiology, Jiann-Ping Hsu College of Public Health, Georgia Southern University.

KWF is an assistant professor in the Journalism and Media Studies Center, the University of Hong Kong.

ZTHT is an assistant professor in the College of Engineering, the University of Georgia.

BS is an MPH student in Jiann-Ping Hsu College of Public Health, Georgia Southern University.

YH is a DrPH student in Jiann-Ping Hsu College of Public Health, Georgia Southern University.

YY is an MS student in the Department of Computer Science, the University of Georgia.

CHC is a PhD student in the Journalism and Media Studies Center, the University of Hong Kong.

## Supplementary Material

Additional file 1 Multilingual abstracts in the six official working languages of the United Nations. Click here for file

Additional file 2 Appendix. Click here for file
