# Supplementary material for: Chinese social media reaction to the MERS-CoV and avian influenza A(H7N9) outbreaks
Source: Infect Dis Poverty. 2013 Dec 20;2:31. doi: 10.1186/2049-9957-2-31 (PMC3878123; doi:10.1186/2049-9957-2-31)
Supplement: Additional file 1 — Multilingual abstracts in the six official working languages of the United Nations. [file 2049-9957-2-31-S1.pdf]

Translation of the abstract into the six official working languages of the United Nations

رد فعل وسائل المواقع الاجتماعية لتفشي أمراض فيروس كورونا والأنفلونزا

إسحق شون هاي فونج، كينج وافو، يوشن يينج، بريدون شيبل، يي هوا،شونج هونج شان، زيون تسو تسي

ملخص

خلفية: ان انطلاق استخدام الانترنت ووسائل المواقع الاجتماعية قد دفع اخصائي الوبائيات باستخدام البيانات المتاحة على الانترنت مثل بيانات جوجول وتويتر لمتابعة مستويات نشاط الأنفلونزا والأمراض المعدية الأخرى في دولة الصين، هناك موقع ويبوالزائع الصيت الخاص بأخصائي الوبائيات وهو مماثل لموقع تويتر يعتمد على الرأي العام الخاص بالصحة العامة حول البيانات المتاحة عبر الموقع، وهو يستخدمت دراسات الموقع كمقياس لرد فعل الصينيين حول تفشي مرضين: فيروس كورونا عام ٢٠١٢ في الشرق الاوسط والأنفلونزا عام ٢٠١٣ بدولة الصين

المنهج: لقد تم البحث باستخدام كلمات بحثيه لبيانات جمعتها جامعة هونج كونج على موقع ويبو تحت مسمى مشروع تليسكوب ويبو ولقد حددت القيم الرئيسية لكل كلمة بحث وقيم ردود الفعل لكل مليون تعليق بعد تفشي المرض ولقد أعلنت المعلومات للعامه

النتائج: أظهرت النتائج ان ردود الفعل كانت قوية للصينيين لكل من المرضيين عبر الانترنت حيث أظهرت ردود فعل المواقع الاجتماعية ترتيب اقوى في الحجم لتفشي وباء الانفلونزا في الصين مقارنه مع تفشي فيروس كورونا خارج الصين

الخلاصة: تشير النتائج إلى أهمية المواقع الاجتماعية لقياس الوعي العام وردود الفعل تجاه المعلومات المعلنة من قبل

Translated from English version into Arabic by Laila Mostafa, through

TRANSLATORS WITHOUT BORDERS

Edited by Dr. Hani Sawami (Georgia Southern University)

中国社交媒体对于中东呼吸症候群冠状病毒感染症和甲型禽流感病毒 H7N9 爆发的反应

Isaac Chun-Hai Fung, King-Wa Fu, Yuchen Ying, Braydon Schaible, Yi Hao, Chung-Hong Chan and Zion Tsz-Ho Tse

摘要

引言：随着互联网和社交媒体的使用率急速上升，流行病学家开始利用谷歌检索数据和推特走势等在线数据，跟踪流感及其它传染病的活动水平。微博是中国版本的推特，是一个极为热门的中国“微型博客”网站。本研究旨在利用微博信息所蕴藏的丰富民意数据，以量度中国人民对两种疾病爆发事件的反应，即2012 年爆发的中东呼吸症候群冠状病毒感染症和2013年在中国爆发的人感染甲型禽流感病毒H7N9事件。

方法：利用香港大学研究项目Weiboscope所搜集得来的微博数据进行关键词检索，为每个关键字设定基线值，设置疫情信息发布后的每百万个发帖设定反应值。

结果：对于两宗暴发事件，中国人在网络上的反应都十分强烈，而社交媒体对发生在中国的甲型禽流感病毒H7N9疫情的反应，远较于发生在中东的冠状病毒感染症事件来得强烈，前者的强度是后者的两个数量级甚至更多。

结论：结果显示，社交媒体可用作量度工具，以之衡量公众对卫生部门所发布的疾病信息的反应，及其关注程度。

Translated from English version into Chinese by Qian Ying-jun, through

CDC CHINESE CENTER FOR DISEASE CONTROL AND PREVENTION
NATIONAL INSTITUTE FOR PARASITIC DISEASES

Edited by Dr. Isaac Chun-Hai Fung (Georgia Southern University) and Ms. Chi-Ngai Cheung (Emory University)

Réaction des réseaux sociaux en Chine face à la pandémie du coronavirus du syndrome respiratoire du Moyen-Orient (MERS-CoV) et de la grippe aviaire A(H7N9)

Isaac Chun-Hai Fung, King-Wa Fu,Yuchen Ying, Braydon Schaible, Yi Hai, Chung-Hong Chan y Zion Tsz-Ho Tse

Résumé

Contexte : Étant donné la monté en flèche de l'utilization de l'internet et des réseaux sociaux, les épidémiologistes ont commencé à exploiter les données en ligne, telles que les données de recherche Google et les tendences Twitter, pour suivre le niveau d'activitéde la grippe et d'autres pathologies infectieuse. Weibo est un site de microblogage équivalent à Twitter qui est extrêmement populaire en Chine. En se basant sur la profusion de données d'opinion publique contenues dans les interventions sur Weibo, cette étude utilisé Weibo pour mesurer les réactions de la population chinoise face à deux pandémies différentes : le coronavirus du syndrome respiratoire du Moyen-Orient (MERS-CoV) en 2012 et la pandémie d'infection humaine en 2013 par la grippe aviaire A(H7N9) en Chine.

Méthodes : Des recherché des mots-clés ont été effectuées sur les données de Weibo recuillies par le projet Weiboscope de l'Université de Hong Kong. Des valeurs de reference ont été attribuées à chaque mot-clés ainsi que des valeurs de reaction par million d'intervention dans les jours qui ont suivi la publication des informations concernant la pandémie.

Résultats : Les résultats montrent que les chinois ont considérablement réagi en ligne face à ces deux pandémies, la réaction des réseaux sociaux étant deux magnitudes plus fortes face à la pandémie de la grippe H7N9, qui avait lieu sur le sol chinois, que par rapport à la pandémie du MERS-CoV, qui se situant loin de Chine.

Conclusions : Les résultats démontrent que les réseaux sociaux peuvent constituer un moyen utile pour sensibiliser et faire reàgir le grande public aux informations de pandémies publiées par les autorités de santé.

Translated from English version into French by Julia Coutinho, through

TRANSLATORS
WITHOUT BORDERS

Edited by Mr. Iurii Bakach (Georgia Southern University)

Реакция в китайских социальных сетях на вспышки коронавируса MERS-CoV и птичьего гриппа A(H7N9)

Исаак Чун-Хай Фунг, Кинг-Ва Фу, Ючен Инг, Брейдон Шайбл, И Хао, Чунг-Хонг Чан и Зион Тц-Хо Тце

Краткий обзор

Предпосылка: В связи с ростом пользования интернетом и социальными сетями, эпидемиологи начали использовать онлайн данные, например, данные Google запросов и Twitter сообщений, для отслеживания уровня активности обсуждения гриппа и других инфекционных болезней. Weibo, являющийся аналогом Twitter, чрезвычайно популярен в Китае. Воспользовавшись богатством информации по общественному мнению, содержащейся в микроблогах Weibo, данное исследование использовало Weibo как меру измерения реакции китайской общественности на две различные вспышки инфекционных заболеваний: вспышку коронавируса MERS-CoV 2012 года и вспышку опасного для человека птичьего гриппа A(H7N9) в Китае в 2013 году.

Методы: Были произведены поиски по ключевым словам в данных микроблога Weibo, собранных в рамках выполняемого Университетом Гонконга проекта «Weiboscope». Были определены базовые значения для каждого ключевого слова и подсчитано количество употреблений на миллион сообщений в дни после опубликования информации о вспышке заболевания.

Результаты: Результаты показывают значительную реакцию китайской общественности в социальных сетях на обе вспышки заболеваний, причем реакция на вспышку гриппа H7N9, произошедшую в Китае, была на два порядка сильнее, чем на вспышку вируса MERS-CoV, имевшую место далеко за пределами Китая.

Заключение: Полученные результаты показывают, что социальные сети могу служить полезным индикатором осведомлённости и реакции общественности на информацию о вспышках заболеваний, публикуемую службами здравоохранения.

Translated from English version into Russian by Alena Hrybouskaya, through

TRANSLATORS WITHOUT BORDERS

Edited by Dr. Yelena Tarasenko (Georgia Southern University) and Mr. Iurii Bakach (Georgia Southern University)

Reacción en las redes sociales de China a los brotes de MERS'CoV y gripe aviar

Isaac Chun-Hai Fung, King-Wa Fu,Yuchen Ying, Braydon Schaible, Yi Hai, Chung-Hong Chan y Zion Tsz-Ho Tse

Resumen

Antecedentes : Como el uso de internet y de las redes sociales se ha disparado, los epidemiólogos han comenzado a utilizar datos en línea, tales como datos de búsquedas en Googleo tendencias de Twitter para registrar los niveles de actividad de la gripa y de otras enfermedades infecciosas. Weibo es una página de microblogs, cuya popularidad en China es comparable a la de Twitter. Gracias a la riqueza de los datos de la opinión pública que se almacenan en sus entradas, este estudio se sirve de Weibo para medir las reacciones de la población china a dos brotes diferentes: el brote en el 2012 del Coronavirus del Síndrome Respiratorio de Oriente Medio (MERS-CoV); y el brote del 2013, en China, de infecciones humanas por gripe aviar de tipo A (H7A9).

Métodos : En el marco del proyecto Weiboscope de la Universidad de Hong Kong se realizaron búsquedas de palabras clave en los datos recopilados de Weibo. Se establecieron valores de partida para cada palabra clave, así como valores de reaccíon por cada millón de entradas publicadas en los días siguientes a la difusión de la información sobre los brotes.

Resultados : Los resultados muestran que el pueblo chino reaccionóde manera significativa a ambos brotes en internet: la reacción en las redes sociales fue de una magnitud dos veces mayor en el brote de gripa de tipo H7N9 que sucedió en China, que en el brote de MERS-CoV, que se produjo lejos del país.

Conclusión : Estos resultados demonstraron que las redes sociales pueden ser un medidor útil de la sensibilización ciudadana y de las reacciones a la información sobre brotes de enfermedades que las autoridades sanitarias publican.

Translated from English version into Spanish by Nothomb, through

TRANSLATORS
WITHOUT BORDERS

Edited by Ms. Claudia Reyes-Garcia (Georgia Southern University)
