# Supplementary material for: Chinese social media reaction to the MERS-CoV and avian influenza A(H7N9) outbreaks
Source: Infect Dis Poverty. 2013 Dec 20;2:31. doi: 10.1186/2049-9957-2-31 (PMC3878123; doi:10.1186/2049-9957-2-31)

Chinese social media reaction to the MERS-CoV and avian influenza A(H7N9) outbreaks

Isaac Chun-Hai Fung[1]*, King-Wa Fu[2], Yuchen Ying[3], Braydon Schaible[4], Yi Hao[4], Chung-Hong Chan[2], Zion Tsz-Ho Tse[5].
* Corresponding author: cfung@georgiasouthern.edu
[1] Department of Epidemiology, Jiann-Ping Hsu College of Public Health, Georgia Southern University, Statesboro, Georgia, USA.
[2] Journalism and Media Studies Center, The University of Hong Kong, Hong Kong Special Administrative Region, China.
[3] Department of Computer Science, The University of Georgia, Athens, Georgia, USA.
[4] Department of Biostatistics, Jiann-Ping Hsu College of Public Health, Georgia Southern University, Statesboro, Georgia, USA.
[5] College of Engineering, The University of Georgia, Athens, Georgia, USA.

Email: Isaac Chun-Hai Fung cfung@georgiasouthern.edu – King-Wa Fu kwfu@hku.hk – Yuchen Ying yegle@uga.edu – Braydon Schaible bs05313@georgiasouthern.edu – Yi Hao yh00111@georgiasouthern.edu – Chung-Hong Chan chanchunghong@hku.hk – Zion Tsz-Ho Tse ziontse@uga.edu

Additional File 2 Appendix

Weibo post data. The data fetched through the user timeline Application Programming Interface (API) consist of the following attributes which were used in the current study: the post message, the latest user profile (including self-reported gender and province of origin/residence), the date and time of posting, whether or not the message is a repost, and the URL of the inline image (if available). If the Weibo message was a repost, all above attributes of both the original and the reposted messages were collected.

Programming language. The Python programming language was used as our tool for data parsing, keyword searching, and statistical analysis of the Weibo weekly files. Python, as a general-purpose scripting language, fits this role because of its built-in Comma-Separated Value (CSV) library that comes with statistical analysis libraries and allows easy manipulation of and access to the metadata of Weibo posts.

Keyword detection algorithm. The searching algorithm was run on a moderate server with an Intel(R) Core(TM) i3-2130 CPU at 3.40GHz and 8G memory. The string-searching algorithm in Python was implemented in C programming language. Python's multiprocessing library was used to process multiple files in parallel to accelerate the search, with a total processing time of 20 minutes for 227,711,963 posts per keyword. The disk read Input Output (IO) speed of the processing server was critical for keyword searching in the huge size of the Weibo raw dataset, which consumed 20% of the total processing time.

Search results display. The search results in CSV format were plotted using Google Chart API to generate static figures as well as interactive Scalable Vector Graphics and Flash charts for data sharing with public health scientists. To make use of Google Chart API, the CSV result files were converted into JavaScript Object Notation (JSON) format, using Python's built-in JSON library. Figure S1 below shows the flowchart of the Keyword Detection Scheme.

Figure S1. Flowchart of the keyword detection scheme. Rectangular blocks are the raw Weibo dataset and the result files. Rhombus blocks are external data and APIs. Round blocks are the python scripts used. Please refer to the main text for more details. API, Application Programming Interface; CSV, Comma-Separated Value; GAR, Global Alert and Response; JSON, JavaScript Object Notation.

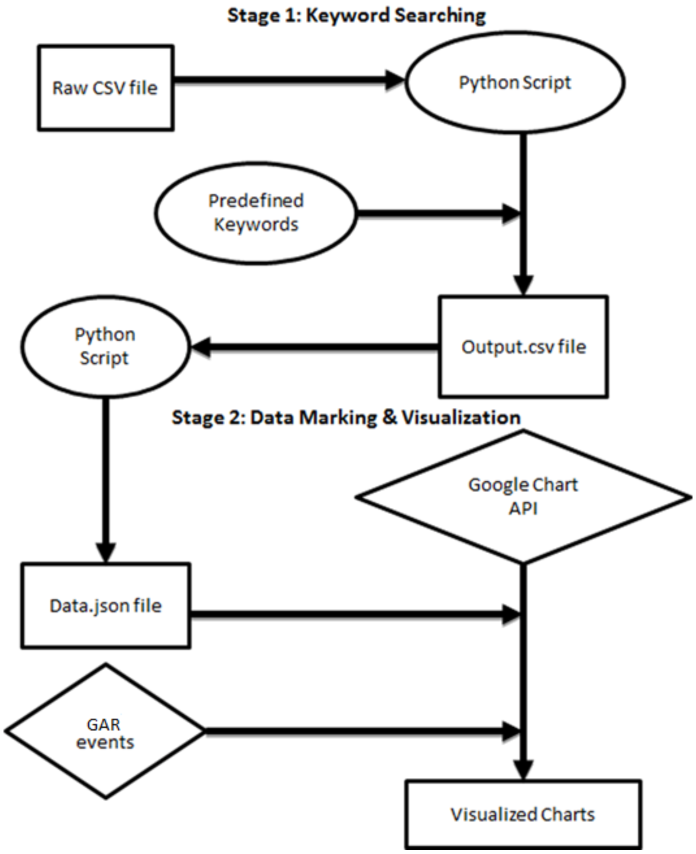

Supplement: Additional file 2 — Appendix. [file 2049-9957-2-31-S2.pdf]
